# A study on the effectiveness and safety of interventional embolization using drug-loaded microspheres in combination with sorafenib and envafolimab for intermediate and advanced renal cell carcinoma

**DOI:** 10.3389/fonc.2025.1558410

**Published:** 2025-05-29

**Authors:** Cheng Chen, Yanfeng Shen, Yanhong Ma, Yingying Zhang, Shuyan Zhang, Na Su, Hefei Guo, Yapeng Guo, Xuehui Zhang, Xianming Liu, Suhua Zhang, Shuai Li, Xin You, Zhiwei Zhang, Xiaoting Duan, Guiying Li

**Affiliations:** ^1^ Department of Oncology, Affiliated Hospital of Hebei Engineering University, Handan, Hebei, China; ^2^ Cath Lab, Affiliated Hospital of Hebei Engineering University, Handan, Hebei, China; ^3^ Department of Respiratory Medicine, Affiliated Hospital of Hebei Engineering University, Handan, Hebei, China; ^4^ Department of Nephrology, Affiliated Hospital of Hebei Engineering University, Handan, Hebei, China

**Keywords:** drug-loaded microsphere interventional embolization, sorafenib, envafolimab, intermediate and advanced renal cell carcinoma, efficacy, safety

## Abstract

**Objective:**

To investigate the effectiveness and safety of drug-loaded microsphere interventional embolization (D-TAE) in conjunction with sorafenib and envafolimab in the management of intermediate and advanced renal carcinoma.

**Methods:**

120 cases of intermediate and advanced renal cell carcinoma cured in the Oncology Department of our hospital from January 2022 to December 2023 were selected. Individuals in the combination group received D-TAE paired with sorafenib and envafolimab. Individuals in the D-TAE group received only D-TAE. The clinical data, clinical efficacy, vascular endothelial growth factor (VEGF), carcinoembryonic antigen (CEA), carbohydrate antigen 125 (CA125), mortality, progression-free survival time (PFS), objective tumor response rate (ORR) and tumor control rate (DCR) and adverse reactions were compared in both groups.

**Results:**

The proportion of individuals with ORR and DCR in the combination group was greatly increased compared to that in the D-TAE group (*P <*0.05). After 1 week and 1 month of treatment, the serum VEGF levels in both groups showed a great decrease compared to pre-treatment levels (*P*<0.05), with the combination group demonstrating notably lower serum VEGF levels than the D-TAE group (*P*<0.05). Following treatment, serum CA125 and CEA levels in both groups experienced a great decrease compared to pre-treatment levels, with the combination group showing notably lower levels than the D-TAE group (*P*<0.05). Additionally, the mortality rate in the combination group was greatly lower than that in the D-TAE group, and the PFS was greatly increased in the combination group compared to the D-TAE group (*P*<0.05). In addition, the observed adverse reactions included gastrointestinal reactions, liver and kidney damage, myelosuppression and rash. Overall, the incidence of adverse reactions in the combination group was greatly decreased than that in the D-TAE group (*P*<0.05).

**Conclusion:**

Drug-loaded microsphere interventional embolization combined with sorafenib and envafolimab has certain efficacy and acceptable safety in treating intermediate and late-stage renal tumor, providing a new treatment option for patients with renal cell carcinoma.

## 1 Preface

Renal cell carcinoma accounts for 80% to 90% of renal malignant tumors and is the most prevalent malignant carcinoma affecting of the kidney and the urinary system (1). Recently, there has been a gradual increase in the occurrence of kidney cancer. Studies have shown that more men than women suffer from kidney cancer, with a ratio of about 2:1; and the incidence rate of kidney cancer is closely related to age (2). The incidence rate of kidney cancer tends to increase with age, peaking between 40 and 55 years. In addition, there are significant regional disparities in the incidence of renal cell carcinoma, with European and American countries showing higher rates than Asian countries, and urban areas having a higher incidence compared to rural areas (3). Most early-stage kidney cancers have no clinical symptoms, so they are easily ignored. Typically, kidney cancer patients are diagnosed through routine physical examinations. Symptoms of kidney cancer typically manifest in the middle and later periods. Current treatments for kidney cancer primarily include surgical resection, targeted therapy, immunotherapy, radiotherapy and chemotherapy. Surgical resection remains the sole potential cure for renal cell carcinoma, but for patients with intermediate and advanced renal cell carcinoma, surgical resection carries higher risks and the scope of resection is larger (4). Targeted therapy and immunotherapy, as non-surgical treatments, can effectively alleviate the disease. However, some patients may develop drug resistance, and other signaling pathways are also involved in the development of cancer, the long-term efficacy remains uncertain (5). Drug-loaded microsphere interventional embolization (D-TAE) is an emerging minimally invasive treatment approach that enables localized chemotherapy of tumors by loading drugs onto microspheres (6, 7). Compared with traditional chemotherapy, D-TAE offers following advantages: slow drug release, which helps reduce toxic side effects; localized administration, leading to decreased systemic toxic reactions; and increased drug concentration at the tumor site, thereby enhancing efficacy. Sorafenib, a broad-spectrum anti-tumor medication, achieves therapeutic results by suppressing tumor angiogenesis and the proliferation of tumor cells (8). Envafolimab is a programmed cell death ligand 1 (PD-L1) inhibitor that recognizes and kills tumor cells by activating the immune system (9). The synergistic anti-tumor effect and enhanced therapeutic outcomes can be achieved through the combined usage of these two drugs. Based upon the aforementioned theory, our goal is to integrate D-TAE therapy into the existing immunotherapy and targeted therapy model. We plan to investigate the combined application of sorafenib, envafolimab and D-TAE in treating intermediate and advanced renal disease. This approach aims to establish an optimal therapy model for patients.

## 2 Materials and methods

### 2.1 Research objects

A total of 155 patients with advanced renal cell carcinoma admitted to the oncology department of our hospital from January 2022 to December 2023 were selected for the study. Participants were categorized into either the combination group and the D-TAE group based on their treatment preferences. The combination group received a treatment regimen consisting of D-TAE in conjunction with sorafenib and envafolimab, while the D-TAE group received D-TAE treatment alone. Prior to enrollment, all individuals and their families executed informed consent documents, and the Ethics Committee granted approval.

Inclusion criteria: patients with inoperable renal cell carcinoma who meet the clinical diagnostic criteria (10); male or female; aged 18~80 years old; staged according to the 8th edition of American Joint Committee on Cancer (AJCC) criteria for renal cell carcinoma, with clinical staging at stage III~IV; Eastern Cooperative Oncology Group (ECOG) performance status score 0~2; normal blood routine and coagulation function; chronic kidney disease (CKD) stage I~II; hypertension classified as stage I~II according to National Cancer Institute-common terminology criteria for adverse events (NCI-CTC AE) 4.0; proteinuria grading standard graded as stage I~II according to NCI-CTC AE 4.0; willing to comply with clinical examination, treatment and follow-up. The specific information is listed in **Table 1** in the form of baseline data.

**Table 1:** Contrast of clinical data between the two groups at baseline ( $\bar{x}$ ± *s*, %).

Clinical information		Combined group (n=60)	D-TAE group (n=60)	*t/*χ ^2^	*P*
Gender	Male	40 (66.67)	45 (75.00)	1.008	0.315
	Female	20(33.33)	15 (25.00)		
Age	/	58.95 ± 12.32	57.77 ± 13.52	0.500	0.618
Tumor size (cm)	/	4.26 ± 1.33	4.61 ± 1.54	1.332	0.185
Renal cell carcinoma (RCC) diagnosis stage	III	38 (63.33)	35 (58.33)	0.315	0.575
	IV	22 (36.67)	25 (41.67)		
Chronic kidney disease (CKD) stage	IV	48 (80.00)	43 (71.67)	1.137	0.286
	V	12 (20.00)	17 (28.33)		
Hypertension stage	I	20 (33.33)	17 (28.33)	0.352	0.553
	II	40 (66.67)	43 (71.67)		
Proteinuria stage	I	11 (18.33)	8 (13.33)	0.563	0.453
	II	49 (81.67)	52 (86.67)		
Eastern cooperative oncology group (ECOG) score	0	8 (13.33)	12 (20.00)	0.984	0.611
	1	25 (28.33)	15 (25.00)		
	2	35 (58.33)	33 (55.00)		
Karnofsky performance status (KPS) score	< 50	9 (15.00)	8 (13.33)	0.575	0.750
	50~60	20 (33.33)	24 (40.00)		
	≥ 70	31 (51.67)	28 (46.67)		
Metastatic sites	Lung	20 (33.33)	22 (36.67)	0.147	0.702
	Bone	10 (16.67)	12 (20.00)	0.233	0.637
	Lymph node	10 (16.67)	13 (21.67)	0.484	0.487
	Liver	6 (10.00)	9 (15.00)	0.686	0.408
	Brain	3 (5.00)	5 (8.33)	0.536	0.464
	Other	7 (11.67)	10 (16.67)	0.617	0.432

Exclusion criteria: individuals with claustrophobia; individuals with severe heart, brain or liver disease, immune system disease or mental or neurological disease who cannot be screened; individuals with severe heart disease, emphysema, asthma or other reasons that hinder screening; individuals with known allergies to contrast media.

### 2.2 Methods

The combination group received treatment with sorafenib, envafolimab in combination with D-TAE, and the D-TAE group was treated with D-TAE alone. Sorafenitol Tosylate Tablets (manufacturer: Bayer AG, Germany, approval number: HJ20160201, specification: 0.2 g) were administered orally at a dosage of 2 tablets per dose, twice daily (11, 12). Envafolimab (Sichuan Silu Kangrui Pharmaceutical Co., Ltd., approval number: S20210046, specifications: 200 mg/bottle) treatment involved a dose of 200 mg administered via subcutaneous injection once every 3 weeks (8, 13). The dosage and medication interval should be adjusted appropriately based on the occurrence of adverse reactions in patients. Both groups continued treatment until disease progression or intolerable adverse reactions occurred. During interventional surgery, the Seldinger method is utilized to puncture and intubate the femoral artery, followed by catheter placement in the renal artery. Renal artery angiography is performed to visualize the area. Subsequently, a detailed analysis of the imaging findings is carried out to determine the location, size, number and supplying arteries of the tumor. Microcatheter ultrasound is also employed. Tumor blood vessels were identified and targeted during the procedure, with the chemotherapy drug epirubicin being administered. Additionally, a combination of poppy seed lipiodol emulsion and drug-loaded microspheres (CaliSpheres; Suzhou Hengrui Callisheng Biomedical Technology Co., Ltd.) was used as the embolic agent. Specifically, drug-loaded microspheres of 100-300 μm in size, containing 40 mg of doxorubicin, were selected for embolization until the tumor staining disappeared. If tumor staining after persists after the consumption of the drug-loaded microspheres and a review of the angiography, further embolization may be performed by adding 300-500 μm microspheres until the tumor is adequately targeted. The blood flow of the main blood vessels was close to stagnant. After the operation, the puncture port was pressure-bandaged and the affected limb was immobilized for 24 h. Upon transfer to the oncology ward, the patient received symptomatic and supportive treatments such as kidney protection, antiemesis therapy, gastric protection, fluid replenishment, and infection prevention measures.

### 2.3 Observation indicators

Clinical data: Register patients’ general data (age, gender, blood pressure, etc.) and clinical characteristics (renal function, cancer metastasis, tumor size, clinical stage, etc.).

Clinical efficacy: Evaluate the patient’s clinical efficacy: Complete response (CR): disappearance of arterial phase improvement and imaging of all identified lesions; Partial response (PR): ≥30% reduction in the sum of the widths of identified lesions; Stable disease (SD): neither meeting the criteria for PR nor progression; disease progression (PD): ≥20% increase in the sum of the widths of identified lesions or appearance of novel lesions. Tumor control rate (DCR) = CR + PR + SD. Objective tumor response rate (ORR) = CR + PR. Thereafter, treatment was continued according to the original plan.

Determination process of vascular endothelial growth factor (VEGF) content in serum: Fasting venous blood was obtained from patients in different treatment groups pre-treatment, on day 1, day 7 and day 30 post-treatment. Subsequently, ELISA was employed for determination following centrifugation.

Serum tumor markers: ELISA was used to detect carcinoembryonic antigen (CEA) and carbohydrate antigen 125 (CA125) in two groups of individuals 30 days before and after treatment.

Follow-up: Through outpatient follow-up or telephone follow-up, as of December 31, 2024, the patient’s mortality, progression-free survival time (PFS) will be recorded.

Adverse reactions: Closely observe the adverse reactions of patients in different treatment groups within 12 weeks.

### 2.4 Statistical techniques

Data were examined by SPSS 21.0. In this study, the quantification data were contrasted using the independent sample *t* test, with ( $\bar{x}$ ± *s*); the comparison of enumeration data between groups in the study was performed using the χ^2^ test, expressed as [cases (%)]. Statistical results deemed *P* < 0.05 as a mathematically great disparity.

## 3 Results

### 3.1 Contrast of clinical data

**Figure 1:**
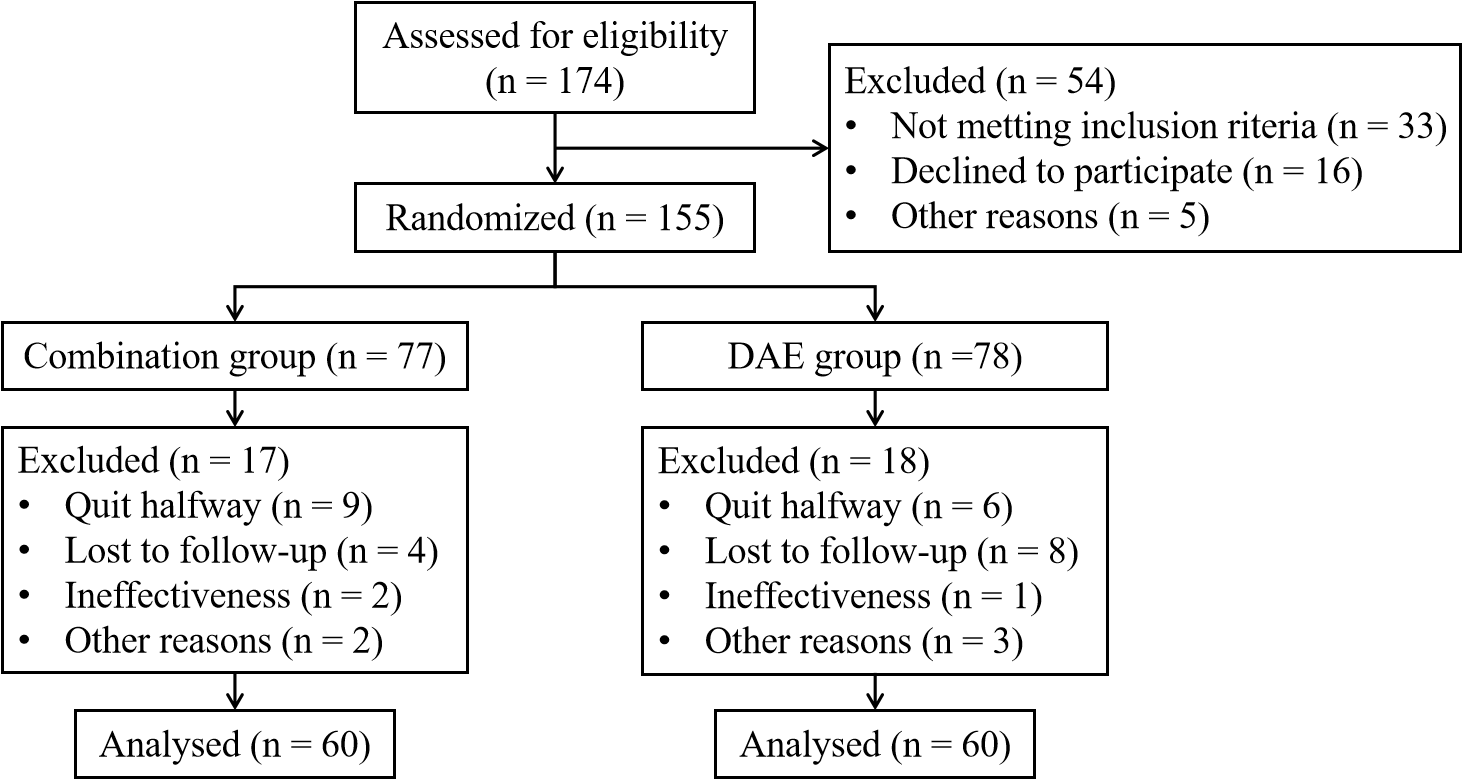
Flowchart of enrollment and allocation of participants and study design.

155 patients who met the inclusion criteria participated in the study. Due to reasons such as quit halfway or losing follow-up, 35 patients were excluded from the study. 120 patients completed the research, with 60 patients in each group respectively. Details are shown in **Figure 1**. There were no discrepancies in clinical data between the two groups in terms of gender, age, tumor size, clinical stage, etc. (*P* > 0.05) (**Table 1**).

### 3.2 Contrast of clinical efficacy

The proportion of individuals achieving ORR and DCR in the combination group was greatly increased compared to that in the D-TAE group (*P <*0.05) (**Table 2**).

**Table 2:** Contrast of clinical efficacy between the two groups [cases (%)].

Group	Number of examples	CR	PR	SD	PD	ORR	DCR
Combination group	60	5 (8.33)	24 (40.00)	9 (15.00)	22 (36.67)	29 (48.33)	38 (63.33)
D-TAE group	60	2 (3.33)	14 (23.33)	11 (18.33)	33 (55.00)	16 (26.67)	27 (45.00)
χ ^2^	/	/	/	/	/	6.647	4.062
*P*	/	/	/	/	/	0.010	0.044

### 3.3 Contrast of serum VEGF levels

1 week and 1 month after treatment initiation, the serum VEGF levels in both groups exhibited a significant decrease compared to pre-treatment levels (*P*<0.05). Furthermore, the serum VEGF levels in the combination group after 1 week and 1 month of treatment were greatly lower than those in the D-TAE group (*P*<0.05) (**Table 3**).

**Table 3:** Serum VEGF levels of the two groups ( $\bar{x}$ ± *s*, ng/L).

Group	Number of examples	Before treatment	Treatment 1 d	Treatment 7 d	Treatment 30 d
Combination group	60	148.32 ± 23.05	147.21 ± 20.69	136.25 ± 19.70* ^#^	113.25 ± 20.59* ^#^
D-TAE group	60	151.02 ± 21.77	150.74 ± 18.96	145.33 ± 22.30* ^#^	129.44 ± 16.47* ^#^
χ ^2^	/	0.660	0.974	2.364	4.756
*P*	/	0.511	0.332	0.020	<0.001

*indicates a great difference compared to the same group before treatment (P < 0.05). ^#^indicates a great difference compared to the same group 1 day after treatment (P < 0.05).

### 3.4 Contrast of tumor marker levels

No discrepancies were observed in serum CA125 and CEA levels between the two groups pre-treatment (*P*>0.05). The serum CA125 and CEA levels were greatly lower post- treatment than pre-treatment. The combination group was greatly lower than the D-TAE group (*P*<0.05) (**Table 4**).

**Table 4:** Contrast of tumor marker levels between the two groups ( $\bar{x}$ ± *s*).

Group	Number of examples	CA125 (U/ml)		CEA (ng/ml)	
		Before treatment	After treatment	Before treatment	After treatment
Combination group	60	366.25 ± 78.35	84.22 ± 12.56*	20.27 ± 5.33	15.21 ± 2.35*
D-TAE group	60	367.36 ± 78.26	144.23 ± 54.24*	20.79 ± 5.46	18.79 ± 2.51*
*t*	/	0.078	8.349	0.528	8.065
*P*	/	0.938	<0.001	0.599	<0.001

Compared to before treatment, *P<0.05.

### 3.5 Contrast of prognosis

The mortality rate of the combination group was greatly decreased compared to that of the D-TAE group, and the PFS was greatly higher (*P*<0.05) (**Table 5**).

**Table 5:** Comparison of prognosis between the two groups ( $\bar{x}$ ± *s*, %).

Group	Number of examples	Mortality rate	PFS (month)
Combination group	60	25 (41.67)	20.12 ± 2.44
D-TAE group	60	36 (60.00)	15.38 ± 3.18
*t/*χ ^2^	/	4.035	9.160
*P*	/	0.045	<0.001

### 3.6 Contrast of adverse reactions

The adverse reactions of patients during the treatment include gastrointestinal reactions, liver and kidney damage, myelosuppression and rash. The occurrence of adverse reactions in the combination group was greatly decreased compared to that in the D-TAE group (*P*<0.05) (**Table 6**).

**Table 6:** Comparison of adverse reactions between the two groups ( $\bar{x}$ ± *s*, %).

Group	Number of examples	Gastrointestinal reactions	Liver and kidney damage	Myelosuppression	Rash	Other	Overall incidence
Combination group	60	4 (6.67)	3 (5.00)	1(1.67)	7 (11.67)	2 (3.33)	12 (20.00)
D-TAE group	60	6 (10.00)	7 (11.67)	3 (5.00)	11 (18.33)	4 (6.67)	23 (38.33)
χ ^2^	/	/	/	/	/	/	4.881
*P*	/	/	/	/	/	/	0.027

## 4 Discussion

Renal cell carcinoma is a rapidly progressive malignant tumor, with approximately one-third of renal cell carcinoma patients have metastatic lesions at the time of diagnosis. Renal cell carcinoma with early stage and regional lymph node metastasis is still mainly treated with surgery, and nearly 25% of patients may experience the development of distant metastasis following radical nephrectomy or nephron-sparing surgery (14). Prior to the recommendation of anti-PD1 immunotherapy drugs for the therapy of renal carcinoma, the prognosis for individuals with distant metastasis was exceedingly bleak, with a 5-year survival rate of only 12% (15, 16). Treatment strategies for advanced metastatic renal cell carcinoma include targeted therapy, immunotherapy, cytoreductive surgery, and palliative radiotherapy to relieve symptoms (17). The employment of immune checkpoint therapeutic antibodies, has significantly improved survival outcomes in advanced metastatic renal cell carcinoma through immunotherapy. The efficacy of anti-PD-1/PD-L1 antibody immunotherapy alone in advanced metastatic renal carcinoma ranges from 25% to 36%. However, the rate of complete tumor elimination rate is only approximately 3% (18, 19).

For patients with intermediate-to-advanced renal cell carcinoma who are unable to undergo or decline surgical resection, interventional treatments such as TAE (renal artery interventional embolization) and ablation therapy have advantages in rapidly reducing tumor burden, inhibiting tumor growth, and alleviating patient pain (20). Most renal cell carcinomas have rich blood supply, which provides a theoretical basis for TAE treatment (21). As a novel drug-loaded embolic agent, drug-loaded microspheres can achieve complete and long-term embolization of tumor blood vessels. Their benefits include sustained drug release and enhanced local drug concentration, making them extensively utilized in clinical practice with favorable clinical outcomes (22). Compared with C-TAE, D-TAE is well tolerated, has better tumor treatment response and longer survival in renal cell carcinoma patients. TAE is a treatment modality for mid-stage and late-stage renal cell carcinoma. Its primary aim is to diminish and obstruct the tumor’s blood supply, causing ischemic and hypoxic necrosis within the tumor. It also induces the production of VEGF and promotes the metastasis, recurrence and spread of the tumor (23, 24). For renal cell carcinomas with a diameter of > 5 cm, there are many blood supplying vessels to the tumor and tumor neovascularization is rapid after surgery, so it is difficult for TAE to completely embolize the tumor at one time. On the other hand, renal cell carcinomas with inadequate blood supply might exhibit reduced sensitivity to ischemia and hypoxia. Therefore, these two types of renal cell carcinoma have higher tumor recurrence and metastasis rates after TAE treatment (25). Sorafenib is a new multi-target anti-tumor drug and a small-molecule multi-kinase inhibitor. *In vivo* and *in vitro* investigations have demonstrated that sorafenib hampers tumor microvessel formation by impeding tumor angiogenesis and cell proliferation, consequently impeding tumor growth (26). It inhibits the activity of several receptor tyrosine kinases, including vascular endothelial growth factor receptor (VEGFR), platelet-derived growth factor receptor (PDGFR), fibroblast growth factor receptor (FGFR)-1, 2, 3 and proto-oncogene tyrosine-protein kinase receptor Ret (Ret), which can play a role in block tumor angiogenesis and reduce the nutrient supply to tumor cells, thereby inhibiting tumor proliferation and metastasis (27). In addition, apoptosis serves as a crucial mechanism through which sorafenib inhibits tumor cell growth. Studies have proven that sorafenib can effectively curb tumor cell growth and prompt apoptosis in human liver cancer cell lines. Furthermore, it demonstrates the ability to impede tumor cell proliferation and induce apoptosis in animal transplanted tumor models. Noteworthy antitumor efficacy has also been observed (28, 29). Since most renal cell carcinoma patients are accompanied by VHL gene mutations, which lead to activation of the HIF pathway and hypoxia, anti-angiogenic drugs are mainly used to directly or indirectly block the downstream signaling pathways of VEGF and its receptors in the treatment of renal cell carcinoma to achieve intervention. The objective of promoting the proliferation of new blood vessels is to target tumor destruction (30). Domestic and foreign scholars are exploring various combination therapies such as PD-1/PD-L1 inhibitors, VEGF inhibitors, and multi-target kinase inhibitors, with the aim of lifting the immune suppression in the tumor microenvironment. Studies have found that when pro-angiogenic factors and anti-angiogenic factors are balanced, abnormal tumor blood vessels transform into a normal phenotype (31, 32). Therefore, the proper use of anti-angiogenic drugs can modify the immunosuppressive tumor microenvironment and directly alleviate hypoxia through various mechanisms to enhance the efficacy of immune checkpoint inhibitors. Envafolimab is a monospecific antibody composed of a single domain antibody (sdAb) and an Fc segment. The molecular weight is half that of the intact antibody, which gives it enhanced penetration while having complete antigen-binding ability (33). Furthermore, Fc-mediated effector functions are attenuated in envafolimab to limit its exposure to the immune system and avoid unintended unnecessary immune responses (34).

The findings of this research demonstrated that drug-loaded microsphere interventional embolization combined with sorafenib and envafolimab was effective in treating intermediate and advanced renal carcinoma. Following treatment, a great reduction in tumor volume was observed, leading to enhanced survival rates. Additionally, there was a substantial decrease in serum tumor markers, and the disease control rate was notably enhanced. This showed that the treatment protocol had a beneficial therapeutic effect on patients with intermediate and advanced renal cell carcinoma. The reason for the analysis was that sorafenib, as a multi-kinase inhibitor of VEGFR-2, VEGFR-3 and others, could fiercely and competitively block the interaction between VEGF to VEGFR-2 and VEGFR-3 and the auto-phosphorylation of the latter, thereby inhibiting tumors. It can improve tumor microcirculation and enhance the immune effect of envafolimab. Upon entering the body, envafolimab can directly block the interaction between PD-L1 and its related ligands, regulating the activity of key signaling pathways, and promoting the body’s resistance to tumor immunity. In terms of safety, this study showed that this treatment protocol was well-tolerated with manageable adverse effects. Sorafenib has been pointed out in previous studies to potentially cause gastrointestinal adverse reactions and rashes (35). Gastrointestinal adverse reactions are also one of the adverse reactions of Envafolimab. Envafolimab may also cause toxicity in bone marrow transplantation and in the liver and kidneys (36). As for D-TAE, its main adverse events may include liver and kidney damage and post-embolism syndrome (37). The adverse effects observed in this study were generally consistent with those previously reported for each treatment. Meanwhile, no patient reported intolerable or withdrew from treatment halfway due to adverse reactions. These findings suggested that the combination of drug-loaded microsphere interventional embolization with sorafenib and envafolimab may become a safe therapeutic approach for treating mid-stage and late-stage renal carcinoma. Although another study pointed out that multi-drug combination therapy may have a greater risk of inducing liver toxicity (38), at least in our study, this phenomenon was not observed. This might be due to the different drugs and intervention methods we use. Sorafenib and envafolimab are distinct anti-tumor medications with unique modes of action. Sorafenib is a multi-target tyrosine kinase inhibitor that can inhibit the growth and angiogenesis of tumor cells. On the other hand, envafolimab is a programmed death protein-1 (PD-L1) inhibitor. It can enhance the killing effect of the immune system on tumor cells. The combined application of two drugs may work together through different mechanisms to improve the therapeutic effect.

In summary, the combination of drug-loaded microsphere interventional embolization along with sorafenib and envafolimab demonstrated promising efficacy and acceptable safety in treating intermediate and advanced renal carcinoma, providing a new treatment option for patients with renal cell carcinoma. While this study provided initial validation of the treatment’s effectiveness and safety, limitations should be considered. Firstly, the small sample size may not fully reflect the overall efficacy and safety of the treatment. Secondly, being a single-center study, the generalizability of the findings could be constrained. Lastly, the short follow-up period limits the assessment of long-term efficacy and survival benefits, indicating the need for further observation.

## Data Availability

The raw data supporting the conclusions of this article will be made available by the authors, without undue reservation.
